# The Direct Testing Effect Is Pervasive in Action Memory: Analyses of Recall Accuracy and Recall Speed

**DOI:** 10.3389/fpsyg.2018.01632

**Published:** 2018-11-13

**Authors:** Veit Kubik, Fredrik U. Jönsson, Monika Knopf, Wolfgang Mack

**Affiliations:** ^1^Department of Psychology, Stockholm University, Stockholm, Sweden; ^2^Berlin School of Mind and Brain, Humboldt-Universität zu Berlin, Berlin, Germany; ^3^Department of Developmental Psychology, Goethe-University, Frankfurt, Germany; ^4^Department of Psychology, Universität der Bundeswehr München, Neubiberg, Germany

**Keywords:** direct testing effect, recall speed, enactment, action memory, distribution-based bifurcation model

## Abstract

Successful retrieval from memory is a desirably difficult learning event that reduces the recall decrement of studied materials over longer delays more than restudying does. The present study was the first to test this direct testing effect for performed and read action events (e.g., “light a candle”) in terms of both recall accuracy and recall speed. To this end, subjects initially encoded action phrases by either enacting them or reading them aloud (i.e., encoding type). After this initial study phase, they received two practice phases, in which the same number of action phrases were restudied or retrieval-practiced (Exp. 1–3), or not further processed (Exp. 3; i.e., practice type). This learning session was ensued by a final cued-recall test both after a short delay (2 min) and after a long delay (1 week: Exp. 1 and 2; 2 weeks: Exp. 3). To test the generality of the results, subjects retrieval practiced with either noun-cued recall of verbs (Exp. 1 and 3) or verb-cued recall of nouns (Exp. 2) during the intermediate and final tests (i.e., test type). We demonstrated direct benefits of testing on both recall accuracy and recall speed. Repeated retrieval practice, relative to repeated restudy and study-only practice, reduced the recall decrement over the long delay, and enhanced phrases’ recall speed already after 2 min, and this independently of type of encoding and recall test. However, a benefit of testing on long-term retention only emerged (Exp. 3), when prolonging the recall delay from 1 to 2 weeks, and using different sets of phrases for the immediate and delayed final tests. Thus, the direct testing benefit appears to be highly generalizable even with more complex, action-oriented stimulus materials, and encoding manipulations. We discuss these results in terms of the distribution-based bifurcation model.

## Introduction

Retrieval practice has attained a great deal of attention as a highly effective study technique for long-term learning (Dunlosky et al., 2013; for a meta-analysis, Rowland, 2014). In recent years, various effects of retrieval have been distinguished (Roediger and Karpicke, 2006b; Roediger et al., 2011). Of most relevance for the current study is the *direct benefit of testing* (or retrieval practice; cf. Karpicke et al., 2014). It refers to the mnemonic effect of retrieving information from memory (for a seminal study, e.g., Bjork, 1975), which appears to reduce the rate of forgetting relative to restudy of information (Roediger and Karpicke, 2006a; Smith et al., 2013; Rowland, 2014). To clarify, taking a test without ensuing feedback, during the learning phase typically leads to inferior memory accuracy after shorter delays compared to an equivalent amount of restudy time; however, this recall advantage vanishes (Putnam and Roediger, 2013, Exp. 1; Jönsson et al., 2014) or even reverses to a test-related recall superiority after longer retention periods (Roediger and Karpicke, 2006a; Keresztes et al., 2013; van den Broek et al., 2013), largely depending on the initial recall success of retrieval-practiced items and the length of the delay (Karpicke and Roediger, 2008; Karpicke and Smith, 2012). In distinction, the *indirect benefit of testing* refers to the enhancing effect of retrieval on subsequent restudy of information (Arnold and McDermott, 2013a,b; Vestergren and Nyberg, 2014; Kubik et al., 2015; Tempel and Kubik, 2017; for a seminal study, e.g., Izawa, 1966).

In the present study, we investigated the direct benefit of retrieval practice. It has been argued that retrieving information from memory is more effortful, compared to the rather fluent restudy practice, and this desirable difficulty of retrieval practice (Bjork, 1994) presumably leads to multiple retrieval routes (McDaniel and Masson, 1985). In that way, retrieval practice promotes long-term retention (*retrieval hypothesis*, e.g., Bjork, 1975; Dempster, 1996). This notion has been elaborated in the *distribution-based bifurcation model* (described later in Section “Introduction”; Halamish and Bjork, 2011; Kornell et al., 2011). Another common account for the direct testing effect is that testing, compared to restudying, seems to foster more efficient semantic binding between cue and target (*semantic elaboration hypothesis*, Carpenter, 2009, 2011; Peterson and Mulligan, 2013; Kubik et al., 2014b), and this partially by activating related extra information (i.e., semantic mediators; Pyc and Rawson, 2010, 2012; Carpenter, 2011). Recently, the episodic context account has been proposed stating retrieval compared to restudy better encodes and updates context information of prior and current learning episodes. This results in enhanced contextual traces that help learners to discriminate the target information better within a reduced search set of retrieval candidates (cf. Karpicke et al., 2014). Up to date, the empirical evidence does not clearly favor one specific theoretical account.

The testing effect has been shown for various materials, such as lists of word pairs (Pyc and Rawson, 2012; Jönsson et al., 2014), prose passages (e.g., Roediger and Karpicke, 2006a), single words (e.g., Carpenter and DeLosh, 2006), or visuospatial information (Carpenter and Pashler, 2007). However, there is scarce evidence of retrieval effects in memory for action events (Kubik et al., 2014b, 2016). Given that memory has likely evolved to remember action-relevant information (Glenberg, 1997), one important venue to enhance our understanding about human learning and memory is to examine action-relevant materials and encoding activities (Roediger and Zaromb, 2010).

To this end, we aimed in the present study to shed light on the robust testing effect under conditions of enhanced encoding via enactment and verbal production within the *paradigm of action memory* (cf. Engelkamp, 1998; Nilsson, 2000; Zimmer et al., 2001; Roediger and Zaromb, 2010; Steffens et al., 2015). Typically, in this paradigm, subjects learn a list of verb–noun phrases (e.g., “to light the candle”) by enacting (i.e., motorically performing) them, observing the experimenter enacting them, or by reading them. A well-established finding is that enacted encoding leads to superior memory accuracy as compared to non-enacted encoding—the so-called *enactment effect* (for seminal papers, see Engelkamp and Krumnacker, 1980; Cohen, 1981; Knopf, 1995). This encoding benefit has been demonstrated under many experimental conditions, most pronouncedly when comparing enacted with read phrases (Nilsson, 2000; Zimmer et al., 2001; Roediger and Zaromb, 2010), and also compared with observed phrases enacted by the experimenter (for a more fine-grained review with more complex action materials, see Steffens et al., 2015).

Previous research demonstrated a testing effect for read action phrases (e.g., “to light the candle”; Kubik et al., 2014b, 2016). However, no such testing effect emerged in terms of reduced forgetting rates when action phrases were enacted (Kubik et al., 2014b), and this irrespective of recall type (Kubik et al., 2016). That is, repeated study–test, relative to repeated study–restudy, practice did not mitigate the recall decrement neither with verb-cued recall of nouns nor with noun-cued recall of verbs. Furthermore, enactment and testing non-additively reduced the rate of forgetting of cued-recall accuracy over a 1-week delay (Kubik et al., 2014b). One possible explanation for these findings is that each study technique already effectively strengthens the association between verb and noun within action phrases, probably in both directions (Carpenter et al., 2006). Such cue–target relational processing, or elaboration of the cue–target association, was proposed as a mechanism to explain both the testing effect (Carpenter, 2009, 2011; Pyc and Rawson, 2012; Peterson and Mulligan, 2013; Kubik et al., 2014b, 2015; Mulligan and Peterson, 2015) and the enactment effect (Kubik et al., 2014a; Steffens et al., 2015; for a review, see Nilsson, 2000; Steffens et al., 2015).

Given the robust testing effect across learning materials and paradigms (Rowland, 2014), the potential lack of this phenomenon in action memory, along with the scarcity of research on the topic (Kubik et al., 2014b, 2015, 2016) motivates further empirical attention as well as methodological consideration. First, as noted by Kubik et al. (2016), previous research used a study design with interleaved testing. That is, restudy opportunities followed testing phases and thereby allowed for the possibility that testing additionally potentiates subsequent restudy (i.e., indirect testing effect; Arnold and McDermott, 2013a,b). In that regard, one aim of the present study was to isolate more clearly the direct from the indirect testing effect on long-term forgetting for action-relevant learning materials. To this end, we did not provide any restudy opportunity following retrieval practice in contrast to previous research (Kubik et al., 2014b, 2015, 2016).

Second, we investigated the direct testing effect on a cued-recall test in terms of both recall accuracy and recall speed—that is, the latency from cue presentation until subjects indicate that they recall the target words (e.g., by pressing a key). Previous accounts primarily focused on the measure of recall accuracy to explain the testing effect in terms of recall decrement or long-term retention. However, recall speed, as a complimentary measure of memory performance, has largely been neglected (but see Keresztes et al., 2013; van den Broek et al., 2013; Racsmány et al., 2018), probably because combined findings of recall accuracy and speed cannot be easily accommodated with previous process-based accounts (cf. van den Broek et al., 2013). However, the distribution-based bifurcation model (Halamish and Bjork, 2011; Kornell et al., 2011) proposes a straightforward explanation for both test-related benefits in terms of the bifurcated distribution of memory strength—an account that is mostly consistent with the majority of previous research findings on the direct testing effect (for a meta-analytic review and evaluation, see Rowland, 2014). To preview, for this reason, we used the distribution-based bifurcation model as a theoretical starting point for our study. However, the aim of the present study was not to explicitly test this framework against other theoretical accounts that, as we acknowledge, may also be feasible to explain the results of our present study (see Section “General Discussion”).

The distribution-based bifurcation model proposes that under retrieval-practiced versus restudied conditions, forgetting may only appear to be mitigated because of the unbalanced re-exposure of the items under restudy and retrieval-practice conditions (if not followed by feedback). Under the testing condition, the items that are correctly recalled gain dramatically in memory strength, whereas items that are not recalled remain unchanged (Bjork and Bjork, 1992; Halamish and Bjork, 2011; Kornell et al., 2011). This results in a bifurcated distribution of memory strength for retrieval-practiced items. In contrast, under the restudy condition, all items are re-exposed and additionally encoded, leading to a parallel boost in memory strength across items, wherefore they remain normally distributed (cf. Halamish and Bjork, 2011). Even assuming equal rates of forgetting, these different item strength distributions would give the memory advantage to restudy conditions after shorter delays (i.e., more studied items will have a memory strength above the threshold) and to testing conditions after longer delays. At least the memory advantage in favor of restudy should plummet with proceeding time. In other words, successfully retrieved relative to restudied items would stay longer above the threshold despite an eventual decrease in memory strength over time. Note that it is reasonable to presume that increases in items’ memory strength are bound to a certain limit; however, they may also exceed the 100% performance level of memory tests as a behavioral proxy. This assumption is, for example, supported by the reliable finding that repeated, compared to single, retrieval can further strengthen items’ memory representations and thereby enlarge the direct testing benefit (cf. Roediger and Karpicke, 2006b).

Given the generality of the direct testing effect for various, even complex study materials, it is reasonable to expect a testing effect to occur for both enactive and verbal encoding of action events. Based on the distribution-based bifurcation model and the above mentioned presumption, we assumed the recall dynamics to occur similarly for both encoding types, though at different levels of memory strength (Kornell et al., 2011). Then, enactive, relative to verbal, encoding can boost the memory strength for all phrases, though to a larger degree. That is, enactive encoding may shift the pre-study memory distributions more upward, reflecting higher memory strength on average. Importantly though, irrespective of encoding condition and memory strength level, successfully recalled phrases should gain more in memory strength than restudied phrases, while non-retrieved phrases remain unchanged. One aim of this study was to test this prediction in action memory with a refined experimental design without restudy opportunity to specifically assess the direct testing effect after verbal and enactive encoding.

Based on the distribution-based bifurcation model, we also expected a testing effect on recall speed. Although more restudied phrases may have a memory strength above the recall threshold during immediate recall, the average memory strength of successfully recalled phrases should be higher, because the processes involved in successful testing are presumably more potent in improving learning. Thus, given that recall latencies reflect more purely memory strength (van den Broek et al., 2013), successfully recalled phrases should be faster recalled than restudied phrases even after short delays. There is only little evidence so far on such an immediate testing effect as only few studies included recall speed (Keresztes et al., 2013; van den Broek et al., 2013; Racsmány et al., 2018). We tested this prediction for the first time in action memory, expecting recall latencies to be shorter for retrieval practiced, as compared to restudied, phrases after both verbal and enactive encoding.

To preview our experimental procedure of this study, we conducted three experiments to examine the direct benefit of testing for enactively and verbally encoded learning materials (e.g., “light the candle”) on recall accuracy and recall speed. In Experiment 1, the direct testing effect was isolated from the indirect testing effect. Subjects encoded a list of action phrases either verbally (i.e., reading them aloud) or enactively (i.e., by motorically performing them). After this initial study (S), participants restudied half of the action phrases twice again either enactively or verbally, and were tested twice on the other half in an intermediate cued-recall test for memory recall (R) (i.e., SSS vs. SRR). Participants were then sequentially provided with nouns (“candle”) as retrieval cues to recall the associated verbs (“to light”). Following both a 2-min and 1-week delay, they received final cued-recall tests, in which they again needed to recall all target words provided with the respective nouns as retrieval cues. Thus, we employed a 2 (practice type: restudy vs. retrieval) × 2 (delay: 2 min vs. 1 week) × 2 (encoding type: verbal vs. enactive) mixed factorial design, with practice type and delay being manipulated within subjects, and encoding type being manipulated between subjects. In Experiment 2, we used the same design as in Experiment 1 but provided verb-cued recall of nouns as intermediate and final tests, instead of noun-cued recall of verbs. In both experiments, we demonstrated the direct testing effect in terms of reduced recall decrement and recall speed, but not enhanced long-term retention. Thus, in Experiment 3, we employed a similar experimental design but with the following critical changes. First, in contrast to Experiments 1 and 2, only half of the retrieval-practiced and restudied phrases were assessed with an immediate final test, and the other half was assessed with the delayed final test. Second, we prolonged the delay from 1 to 2 weeks. Third, we implemented two initial study phases (i.e., SSRR vs. SSSS) to decrease the differential exposure advantage for restudied with phrases. Fourth, we added a condition without any interim activity (i.e., SS). As a result, we obtained a cross-over interaction between practice type and delay as well as a long-term recall benefit, with encoding type not significantly moderating this direct testing benefit.

## Experiment 1

### Methods

#### Subjects

We pre-determined a sample size of 24 subjects for each encoding group that was, however, not based on an *a priori* power calculation. Instead of a *post hoc* power calculation for non-significant results, we provided 95% confidence intervals (CIs; cf. Colegrave and Ruxton, 2003). In total, 48 German young adults were individually tested (*M* [*SD*] age, 32.521 [9.065]; 27 females; working-memory capacity, 58.583 [12.005], for a description of the operation-span task, see Unsworth et al., 2005). Their data were included in the final analysis. Three additional subjects were tested but excluded, because no data were available at one of the final tests. Subjects from this convenience sample were all native German speakers and participated voluntarily or in return for course credits. They were randomly assigned to the two groups of encoding type (enactive vs. verbal), with the restriction of obtaining a similar gender ratio (enactive: 13 females; verbal: 14 females). Similar subjects characteristics were achieved between groups, such as mean age (enactive: 31.125 [10.079]; verbal: 33.917 [7.890]), *U* = 247.500, *p* = 0.408, *r*_rb_ = 0.141, 95% CI [-0.186, 0.439], and working-memory capacity (enactive: 56.833 [9.990]; verbal: 60.333 [13.723]), *U* = 199.000, *p* = 0.068, *r*_rb_ = 0.309, 95% CI [-0.011, 0.571].

#### Design

A 2 (practice type: restudy vs. retrieval) × 2 (delay: short vs. long) × 2 (encoding type: verbal vs. enactive) mixed factorial design was applied. Practice type and delay were manipulated within-subjects, and encoding type was manipulated between-subjects. The main dependent variables were recall accuracy, delay-contingent recall decrement,^1^ and recall speed. Concerning recall speed, we considered only item-specific response latencies^2^ of correctly recalled targets (i.e., mean response latencies to press the spacebar in seconds [s] at the immediate final test^3^). Both measures assess the direct testing effect independent of external factors, such as the size of the restudy advantage after the short delay.

#### Materials

Stimuli were 40 German action (i.e., verb–noun) phrases (e.g., “light a candle”) selected from a normed item pool of action phrases (Mohr et al., 1991; provided and used in Steffens et al., 2006; Exp. 1). They comprised one verb and one noun, were two to four words long, and did not include body parts as objects (e.g., “lift an arm”). The action phrases were divided into two lists, each comprising 20 action phrases of high association strength and 20 action phrases of low association strength. We counterbalanced the assignment of the two lists and item sets evenly to practice type conditions (restudy vs. retrieval) across subjects, separately for encoding groups. We assessed working-memory capacity by assessing the operation span (i.e., mean number of items recalled in the correct position across set sizes, cf. Unsworth et al., 2005).

#### Procedure

Subjects underwent an initial learning session, an immediate final test session after 2 min, and a delayed final test session after 1 week. In the initial learning session, they studied (S) 40 action phrases. During the two subsequent practice phases, half of the action phrases were practiced twice by restudy (i.e., restudy condition, SSS), and the other half was practiced twice by retrieval (R) in an intermediate cued-recall test (i.e., retrieval condition, SRR); they were displayed in a random, mixed order. Subjects completed a 30-s arithmetic filler task (i.e., judging the correctness of mathematical equations) between practice phases in order to prevent recency effects. During each study and restudy trial, one action phrase was presented for 8 s in a random order, separated by a 1-s interstimulus interval. Depending on the encoding group, subjects were asked in each study or restudy phase to read the action phrase aloud (i.e., verbal encoding) or motorically performing it without any physical object (e.g., a candle) at hand (i.e., enactive encoding). The experimenter was in the room to secure that the subjects complied with the instructions. During each of the test trials, the noun (“a candle”) of the previously studied action phrase (“light a candle”) was displayed as the retrieval cue for max. 8 s, one at a time, or until the subjects pressed the SPACE key to indicate that they do remember the target verb (“light”). The remainder of the 8 s were then provided to type the target verb on the computer keyboard. Response latency was measured as the time from cue presentation until pressing SPACE. The presentation order of the phrases across practice type conditions was uniquely randomized for each subject and phase.

After the learning phase, a 2-min-long arithmetic filler task was given, followed by the immediate test session. Subjects returned after 1 week for the delayed test session. In both test sessions, subjects received a final cued-recall test for all action phrases in a uniquely random order. The procedure of intermediate and final tests was identical. The experiment ended with the automated operation span task.

#### Scoring and Analyses

Subjects’ responses were scored as correct if the original verbs target (e.g., “light”) from the action phrases (e.g., “light a candle”) was entered on the keyboard. We reported the results based on this strict evaluation criterion. Similar results were obtained when evaluating the data following a more lenient criterion that scores also synonymous verbs as correctly recalled. To analyze recall accuracy and recall speed as a function of practice type, delay, and encoding type, we conducted mixed-factorial analyses of variance (ANOVA). To follow-up significant interactions, we conducted simple-effects analyses. In cases when the assumption of sphericity was violated, the reported numbers were calculated using a Huynh–Feldt correction. Population-based effect sizes (omega squared, 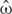
^2^) were reported and an alpha level of 0.05 was used. Selectively, we reported planned comparisons between specific conditions or experimental groups based on one-sided Student *t*-tests (with Cohen’s *d* as effect-size measures) or equivalent non-parametric statistics when the assumptions of normality and/or homoscedasticity were violated. To control for the family-wise error rate, the alpha level was Bonferroni-corrected for planned comparisons. The materials, data and analysis scripts are available on the Open Science Framework.^4^

### Results and Discussion

**FIGURE 1:**
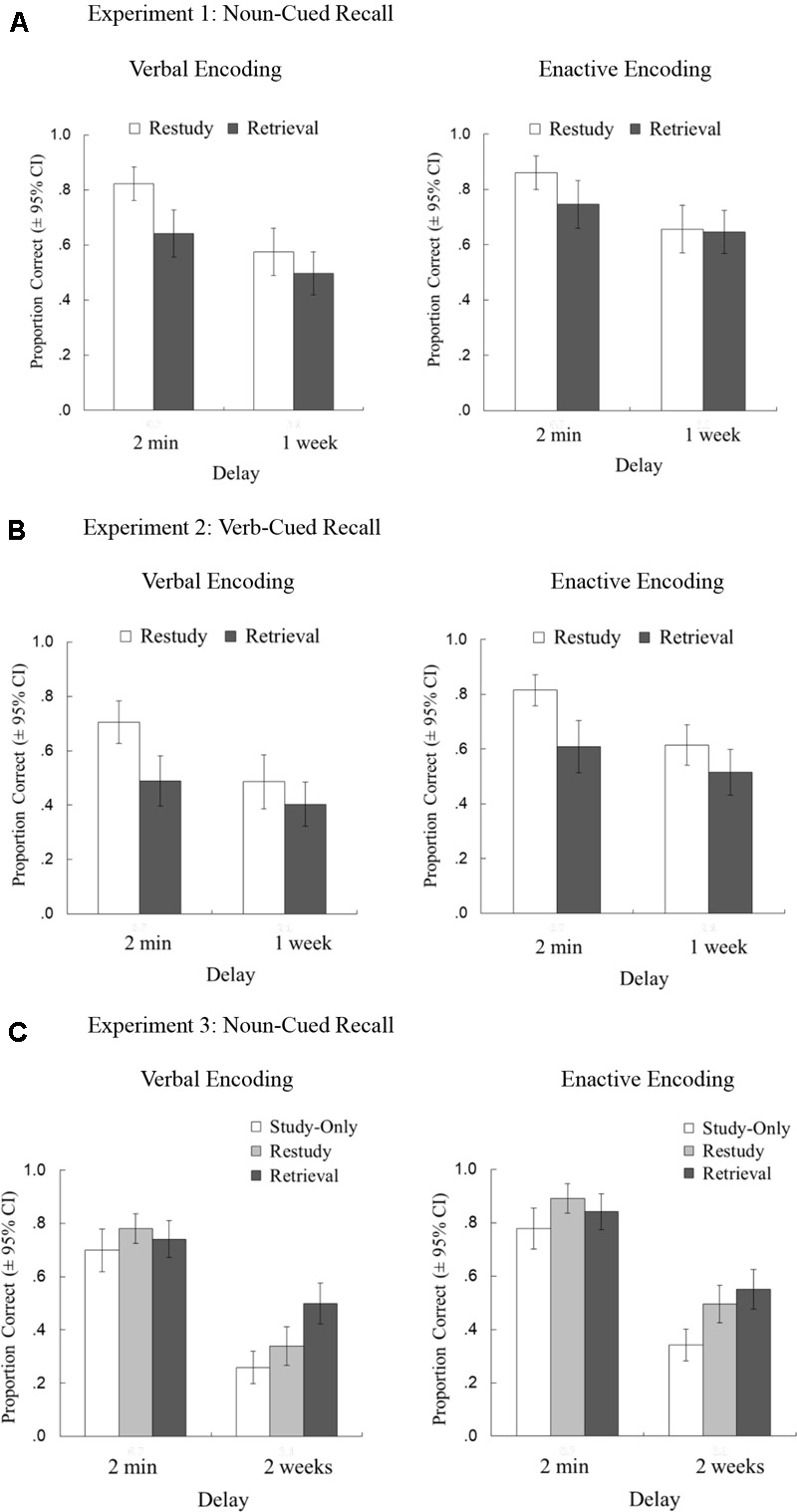
Final recall accuracy (mean proportion correct and 95% confidence intervals [CIs]) for studied action phrases, as a function of practice type delay and encoding type, separately shown for Experiment 1 [**(A)** noun-cued recall], Experiment 2 [**(B)** verb-cued recall], and Experiment 3 [**(C)** noun-cued recall].

Figure 1 illustrates the results on recall accuracy after short and long delays.

#### Recall Accuracy

As can be seen in Figure 1A, retrieval-practiced action events were less recalled than those restudied after the short delay; however, this recall advantage in favor of restudy practice diminished over 1 week, similarly for both encoding groups. A mixed factorial ANOVA demonstrated a main effect of practice type, *F*(1, 46) = 19.768, *p* < 0.001, 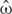
^2^ = 0.065 (restudy: 0.729 [0.176]; retrieval: 0.633 [0.195]), and a marginal effect of enactive type (enactive: 0.727 [0.150]; verbal: 0.634 [0.221]), *F*(1, 46) = 3.900, *p* = 0.054, 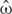
^2^ = 0.057. There was also a significant main effect of delay, *F*(1, 46) = 107.427, *p* = 0.001, 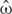
^2^ = 0.204, indicating that recall accuracy decreased after 1 week (short: 0.768 [0.173]; long: 0.594 [0.198]). More importantly, we observed a significant practice type × delay interaction, *F*(1, 46) = 25.785, *p* < 0.001, 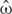
^2^ = 0.023, indicating that testing reduced the recall decrement from short to long delays. That is, the immediate recall advantage of restudied over retrieval-practiced phrases, *W* = 895.500, *p* = 0.001, *r_rb_* = 0.523, 95% CI [0.250, 0.719], was diminished after 1 week on long-term retention, *t*(47) = 1.793, *p* = 0.079, *d* = 0.259, 95% CI [-0.030, 0.545]. Critically, the effect of practice type was not significantly moderated by encoding type, as demonstrated by a non-significant practice type × encoding type interaction, *F*(1, 46) = 2.392, *p* = 0.129, 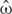
^2^ = 0.005, and a non-significant practice type × delay × encoding type, *F*(1, 46) < 0.001, *p* > 0.999, 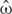
^2^ < 0.001. There was no significant interaction effect between encoding type and delay, *F*(1, 46) = 1.699, *p* = 0.199, 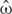
^2^ = 0.002.

#### Proportional Recall Decrement

**FIGURE 2:**
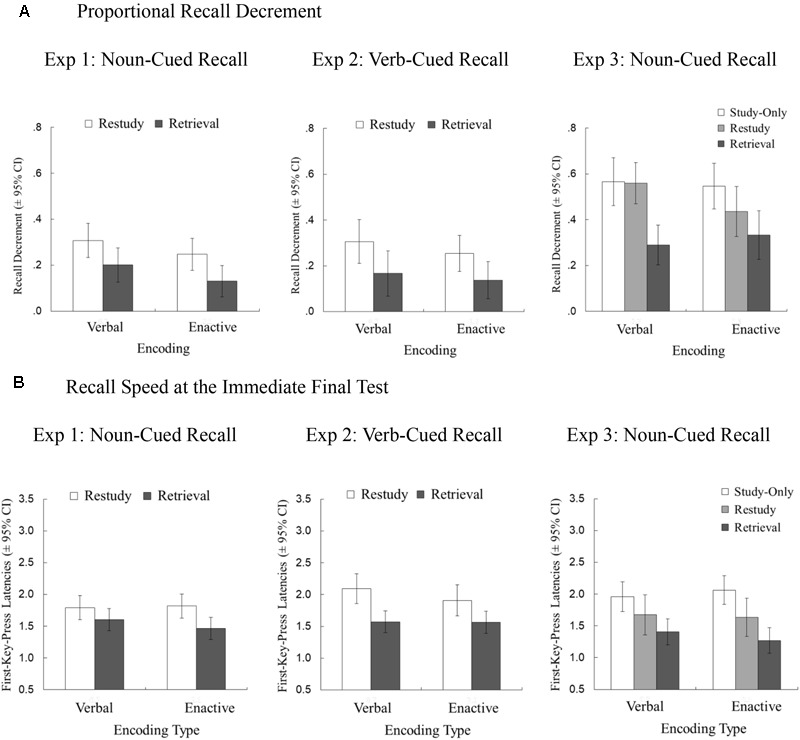
Proportional recall decrement (mean proportion of decreased recall accuracy over the long delay and 95% CIs) and recall speed (i.e., mean first-key-press latencies in s and 95% CIs at the immediate final test for the correctly recalled target words), as a function of practice type (retrieval/restudy/study-only) and encoding type (enactive/verbal). Proportional recall decrement **(A)** and recall speed **(B)** are shown separately for Experiment 1 (i.e., noun-cued recall of verbs), for Experiment 2 (i.e., verb-cued recall of nouns), and for Experiment 3 (i.e., noun-cued recall of verbs).

Figure 2A shows the proportional recall decrement as a function of practice type and encoding type. Retrieval practice (*M* = 0.166, *SD* = 0.164), compared to restudy practice (*M* = 0.278, *SD* = 0.181), led to a reduced recall decrement, as indicated by a main effect of practice type, *F*(1, 46) = 16.710, *p* < 0.001, 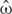
^2^ = 0.090. However, the recall decrement did not differ between enacted phrases (*M* = 0.189, *SD* = 0.156) and read-aloud phrases (*M* = 0.254, *SD* = 0.188), as shown by a non-significant main effect of encoding type, *F*(1, 46) = 2.421, *p* = 0.127, 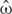
^2^ = 0.029. More importantly, there was no significant practice type × encoding type interaction, *F*(1, 46) = 0.035, *p* = 0.853, 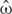
^2^ < 0.001, indicating that the direct testing effect did not reliably differ between enactive and verbal encoding.

#### Recall Speed

Figure 2B shows recall speed as a function of practice type, delay, and encoding type. As predicted, verb targets were reliably faster accessed for retrieval-practiced phrases (*M* = 1.534, *SD* = 0.419) than for restudied phrases (*M* = 1.806, *SD* = 0.461), as indicated by a main effect of practice type, *F*(1, 46) = 30.597, *p* < 0.001, 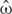
^2^ = 0.085. There was no significant main effect of encoding type, *F*(1, 46) = 0.227 *p* = 0.636, 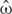
^2^ < 0.001, and there was no significant encoding type × practice type interaction effect, *F*(1, 46) = 2.851 *p* = 0.098, 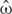
^2^ = 0.006, indicating that the advantage of retrieval practice, compared to restudy practice, in recall speed did not significantly differ between verbal and enactive encoding.

In sum, the testing effect was demonstrated for action events in terms of both reducing the recall decrement over 1 week and enhancing recall speed, and this largely independent of whether action phrases were read aloud or enacted. However, we did not observe any test-related recall advantage after 1 week, but the restudy advantage was reduced from short- to long-term retention. This finding is in parts due to the fact the subjects failed to recall, and thereby to re-experience only 68.229% (*SD* = 22.061%) of the tested items during the second intermediate test; that is, 74.375% (*SD* = 13.856%) of the enacted phrases and 62.083% (*SD* = 26.902%) of the read-aloud phrases. In comparison, 100% of the restudied phrases were re-experienced (for further elaboration, see Section “General Discussion”). Proportional recall decrement and recall speed were more sensitive measures to reflect the direct testing effect.

## Experiment 2

Given the novelty of this results pattern, and that enactment was previously shown to preempt the testing effect in terms of a reduced recall decrement when restudy- and retrieval-practice phases were interleaved (Kubik et al., 2014b, 2016), the primary goal of Experiment 2 was to conceptually replicate the findings of Experiment 1 with verb-cued recall as the intermediate and final tests. Instead of the nouns, we provided verbs (e.g., “light”) as retrieval cues, and the subjects needed to recall the respective target nouns (e.g., “a candle”) during intermediate and final memory tests. All other aspects of the procedure were identical to Experiment 1. Based on previous findings that the retrieval direction (noun-cued recall of verbs vs. verb-cued recall of nouns) has no moderating influence (Kubik et al., 2015, 2016), we predicted to find a retrieval-practice effect on the recall decrement using verb-cued recall tests. This replication effort supports the current emphasis on results’ replicability (Pashler and Wagenmakers, 2012; Open Science Collaboration, 2015).

### Methods

#### Subjects

35 young adults (*M* [*SD*] age, 26.286 [5.154], working-memory span, 57.743 [11.197], 18 females) voluntarily participated in this experiment until the end of the term, and their data were included in the final data analysis. Instead of *post hoc* power calculation for non-significant results, we provided 95% CIs (cf. Colegrave and Ruxton, 2003). One additional subject was tested but excluded, as there were no data available at the delayed final test due to a technical error. Subjects were randomly assigned to each of the two encoding groups (enactive vs. verbal), with the restriction of obtaining a similar gender ratio (enactive: 9 females; verbal: 9 females). Between groups, no significant differences in subjects’ characteristics were obtained, such as mean age (enactive: 25.235 [4.039]; verbal: 27.278 [5.969]), *U* = 128.000, *p* = 0.416, *r_rb_* = 0.163, 95% CI [-0.220, 0.503], and working-memory capacity (enactive: 55.353 [13.271]; verbal: 60.000 [8.595], *U* = 124.500, *p* = 0.355, *r_rb_* = 0.186, 95% CI [-0.197, 0.520]).

#### Design, Materials, and Procedure

The methodological aspects were identical to Experiment 1, with the exception that verb-cued recall was given during the intermediate and final test phases. That is, subjects saw the verbs (e.g., “light”) of the action phrases (e.g., “light a candle”), one at a time, as retrieval cues and were instructed to recall the respective noun targets (e.g., “a candle”). Subjects’ responses were scored as correct if the original noun targets were entered on the keyboard.

### Results and Discussion

#### Recall Accuracy

As can be seen in Figure 1B, retrieval-practiced action events were less recalled than those restudied after the short delay; however, this recall advantage in favor of restudy practice diminished over 1 week. A mixed-factorial ANOVA demonstrated main effects of practice type, *F*(1, 33) = 31.156, *p* < 0.001, 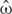
^2^ = 0.161 (retrieval: 0.504 [0.179]; restudy: 0.655 [0.185]), and encoding type (enactive: 0.638 [0.174]; verbal: 0.521 [0.190]), *F*(1, 33) = 5.191, *p* = 0.029, 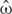
^2^ = 0.107. Recall accuracy decreased reliably over 1 week, as shown by a significant main effect of delay, *F*(1, 33) = 72.612, *p* < 0.001, 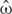
^2^ = 0.180 (short: 0.654 [0.176]; long: 0.505 [0.187]). More importantly, we observed a significant practice type × delay interaction, *F*(1, 33) = 23.606, *p* < 0.001, 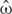
^2^ = 0.035, indicating that testing reduced the recall decrement from short to long delays. That is, the immediate recall advantage of restudied over retrieval-practiced phrases, *t*(34) = 8.908, *p* < 0.001, *d* = 1.506, 95% CI [1.014, 1.987], was diminished after 1 week on long-term retention, *t*(34) = 2.682, *p* = 0.011, *d* = 0.453, 95% CI [0.102, 0.799]. Critically, the effect of practice type was not moderated by encoding type, as demonstrated by a non-significant practice type × encoding type interaction, *F*(1, 33) = 0.003, *p* = 0.957, 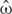
^2^ < 0.001, and a non-significant practice type × delay × encoding type, *F*(1, 33) = 0.311, *p* = 0.581, 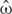
^2^ < 0.001. There was no interaction effect between encoding type and delay, *F*(1, 33) = 0.026, *p* = 0.872, 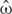
^2^ < 0.001, indicating that the recall decrement over the 1-week delay did not differ between enactive and verbal encoding.

#### Proportional Recall Decrement

A significant main effect of practice type, *F*(1, 33) = 12.839, *p* = 0.001, 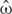
^2^ = 0.104, indicated a testing effect, with retrieval-practiced phrases (*M* = 0.152, *SD* = 0.163) decreasing less in recall accuracy than restudy-practiced phrases (*M* = 0.280, *SD* = 0.199; see Figure 2A). Similarly as for noun-cued recall in Experiment 1, the recall decrement did not differ between enacted phrases (*M* = 0.196, *SD* = 0.183) and read-aloud phrases (*M* = 0.236, *SD* = 0.179), as shown by a non-significant main effect of encoding type, *F*(1, 33) = 0.644, *p* = 0.428, 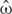
^2^ < 0.001. More importantly, there was no significant practice type × encoding type interaction, *F*(1, 33) = 0.092, *p* = 0.764, 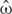
^2^ < 0.001, indicating that the direct testing effect did not reliably differ in size as a function of encoding type.

#### Recall Speed

Consistent with Experiment 1, noun targets were faster accessed for retrieval-practiced action phrases (*M* = 1.568 s, *SD* = 0.350) than for restudied action phrases (*M* = 1.999 s, *SD* = 0.480), *F*(1, 33) = 44.849, *p* < 0.001, 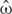
^2^ = 0.202. This test-related advantage in recall speed did not differ as a function of encoding type, as indicated by a non-significant practice type × encoding type interaction, *F*(1, 33) = 1.899, *p* = 0.177, 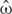
^2^ = 0.005. Recall speed did not differ between enacted phrases (*M* = 1.736 s, *SD* = 0.344) and verbally encoded phrases (*M* = 1.832 s, *SD* = 0.486), as indicated by a non-significant main effect of encoding type, *F*(1, 33) = 0.550, *p* = 0.464, 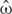
^2^ < 0.001.

To conclude, the results of Experiment 2 also demonstrated an interaction effect between delay and practice type on final recall accuracy, such that testing, compared to restudy, reduced the recall decrement over the long delay. In addition, there was evidence for a testing effect on recall speed, that is, recalled phrases were faster retrieved than restudied phrases, even after the short delay. Both findings were obtained independently of encoding group. However, in contrast to the majority of prior studies (Roediger and Karpicke, 2006a; Toppino and Cohen, 2009; Kornell et al., 2011) and similar to Experiment 1, we did not find a cross-over interaction effect that would result in a testing advantage on long-term retention. One reason could be that all restudied phrases were re-encoded twice during practice phases, while retrieval-practiced phrases were only re-experienced twice when they were successfully recalled. In fact, similar to Experiment 1, subjects failed to recall, and only re-experienced 52.714% (*SD* = 19.378%) of the tested items during the second intermediate test; that is, 58.529% (*SD =* 17.209%) of the enacted phrases, and 47.222% (*SD =* 20.163%) of the read-aloud phrases, compared to 100% of the restudied phrases were re-experienced. The delay of 1 week may thus have been too short, at least for action events, to revert this immediate restudy advantage to a testing effect on long-term retention. In addition, in Experiments 1 and 2 (and also in Kubik et al., 2014b, 2016), all phrases were tested after both the short and long delays. Consequently, the immediate test event likely also strengthened the memory trace of restudied phrases, and thereby reducing the size of a potential testing advantage in long-term retention and the practice type × delay interaction.

## Experiment 3

In Experiment 3, we employed a design affording a clearer comparison between the effects of retrieval vs. restudy practice in that only half of the retrieval-practiced and restudied phrases were assessed with an immediate final test, and the other half was assessed with the delayed final test. In addition, we prolonged the delay to 2 weeks and decreased the differential exposure advantage for restudied phrases by implementing two initial study phases (i.e., SSRR vs. SSSS) as well as added a condition without any interim activity (i.e., SS). Given these design changes, we predicted the delay-contingent testing effect to reverse into superior long-term retention irrespective of encoding type and tested this directional hypothesis by conducting one-sided *t*-tests or the non-parametric alternative.

### Method

#### Subjects

As we were specifically interested in long-term retention of enacted action events, and previous research did not find any testing effect on this measure, we pre-determined a somewhat larger sample size of 28 subjects each encoding group that was, however, not based on an *a priori* power calculation. Instead of *post hoc* power calculation for non-significant results, we provided 95% CIs (cf. Colegrave and Ruxton, 2003). In total, 68 younger adults voluntarily participated in this experiment, and were individually tested at Stockholm University, Sweden. Subjects from this convenience sample were all native Swedish speakers. They were randomly assigned to each of the two encoding groups (enactive vs. verbal). Data of additional 10 subjects were collected but not included in the final analyses for the following reasons: (i) they did not come back after the 2-week interval (*n* = 6); (ii) subjects had already participated in a highly similar study in our lab on the testing effect with the same action materials (*n* = 1); and (iii) there were no data available at one of the intermediate or final tests due to a technical error (*n* = 3). The final sample consisted of 58 subjects (*M* [*SD*] age, 24.000 [4.675], 41 females). Between groups, there was a similar gender ratio (enactive: 20 females; verbal: 21 females), and a descriptive, though non-significant difference in mean age emerged (enactive: 22.800 [3.242]; verbal: 25.286 [5.616]), *U* = 295.500, *p* = 0.052, *r*_rb_ = 0.296, 95% CI [0.006, 0.541]) that was inconsequential; controlling for age in the critical analyses of variance did not considerably change the reported results.

#### Design, Materials, and Procedure

The methodological aspects were similar to Experiment 1, using a noun-cued recall of verbs in intermediate and final tests, but with the following main differences. First, 48 Swedish action phrases were selected from the normative study of Kormi-Nouri (1995) comprising 24 action phrases of high association strength and 24 action phrases of low association strength. Second, the experimental design was extended. Subjects learned action phrases in two initial study phases (instead of only one), in which questions of study ease were provided after studying the individual phrases. These results were not the focus of this investigation, and not reported here. Third, there were three (instead of two) types of practice: in addition to practice conditions of repeated retrieval (SSRR) and repeated restudy (SSSS), we provided a study-only condition with no interim phases (SS) as a further control condition. The selected action phrases of each list were evenly divided in three sets of 16 items (comprising 8 phrases of both high and low association strength) that were assigned to the practice-type conditions (retrieval, restudy, vs. study-only) in a counterbalanced fashion across subjects, equivalently for both encoding-type groups. Fourth, half of the action phrases, proportionally distributed across practice conditions, were to be recalled in a final memory test after the short delay, and the other half of the action phrases were to be recalled after the long delay. The assignment of the two item lists to the delays was counterbalanced across subjects. To ensure a similar short delay for the study-only condition, the immediate final test was placed at the time as the intermediate tests for the restudy and retrieval conditions during the learning phase. After the 2-week delayed tests, subjects received two additional cued-recall tests with feedback. For the sake of brevity and focus, the results of these tests were not reported here. Fifth, at the beginning of the experiment, a psychophysical test of simple motor reaction time was given to take into account individual differences in motor reaction times that may conflate individual’s retrieval latencies (Obermeyer et al., 2012). Subjects simply needed to press, with each hand separately (2 × 60 trials), the target button once a stimulus (a black dot, 2° visual angle) on a gray background appeared. To obtain purified cognitive latency measures of individual’s recall speed data, each subject’s motor reaction time mean was assessed for both hands, and subsequently subtracted from the individual’s retrieval latencies.

### Results and Discussion

#### Recall Accuracy

Analyses on recall accuracy showed a main effect of encoding type, demonstrating that enacted phrases (*M* = 0.650, *SD* = 0.167) were better recalled than read-aloud phrases (*M* = 0.553, *SD* = 0.198), *F*(1, 56) = 8.132, *p* = 0.006, 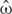
^2^ = 0.110, as well as a main effect of practice type, *F*(1.857, 104.012) = 36.675, *p* = 0.001, 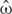
^2^ = 0.135 (retrieval: 0.658 [0.192]; restudy: 0.627 [0.170]; study-only: 0.520 [0.186]). Recall accuracy decreased reliably over 2 weeks, as shown by a significant main effect of delay, *F*(1, 56) = 470.446, *p* < 0.001, 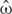
^2^ = 0.629 (short: 0.789 [0.179]; long: 0.414 [0.186]). Critically, we observed a cross-over practice type × delay interaction, *F*(2, 112) = 11.298, *p* < 0.001, 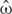
^2^ = 0.054. Simple-effect analyses indicated that there is a significant main effect of practice type after the short delay, *F*(2, 112) = 7.611, *p* < 0.001, and after the long delay, *F*(2, 112) = 34.251, *p* < 0.001. That is, the immediate recall advantage of restudied over retrieval-practiced phrases, *W* = 349.500, *p* = 0.041, *r*_rb_ = 0.591, 95% CI [0.366, 0.751] (one-tailed), reverted into a testing advantage on long-term retention, compared to both restudied phrases, *t*(57) = 3.528, *p* < 0.001, *d* = 0.463, 95% CI[0.190, 0.732] (one-tailed), and study-only phrases, *W* = 1213.500, *p* < 0.001, *r*_rb_ = 0.418, 95% CI [0.149, 0.630] (one-tailed). Importantly, the effect of practice type was not moderated by encoding type, as indicated by a non-significant practice type × encoding type interaction, *F*(1.857, 104.012) = 1.770, *p* = 0.178, 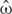
^2^ = 0.003, and a non-significant practice type × delay × encoding type, *F*(2, 112) = 0.745, *p* = 0.477, 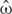
^2^ < 0.001. There was no encoding type × delay interaction effect, *F*(1, 56) < 0.001, *p* = 0.984, 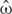
^2^ < 0.001, indicating that enactive, compared to verbal, encoding did not significantly reduce the recall decrement after the long delay of 2 weeks.

#### Proportional Recall Decrement

A 3 (practice type: retrieval, restudy, vs. study-only) × 2 (encoding type: verbal vs. enactive) mixed ANOVA showed a significant main effect of practice type, *F*(2, 112) = 14.157, *p* < 0.001, 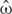
^2^ = 0.125. Planned comparisons revealed that repeated retrieval led to a decreased recall decrement (*M* = 0.312, *SD* = 0.289) compared to both repeated restudy (*M* = 0.497, *SD* = 0.237), *W* = 1192.500, *p* < 0.001, *r*_rb_ = 0.394, 95% CI [0.120, 0.612] (one-tailed), and study-only (*M* = 0.556, *SD* = 0.270), *t*(57) = 5.218, *p* < 0.001, *d* = 0.685, 95% CI [0.396, 0.969] (one-tailed). Similarly as in Experiments 1 and 2, the recall decrement did not differ between enacted phrases (*M* = 0.439, *SD* = 0.234) and read-aloud phrases (*M* = 0.472, *SD* = 0.296), as shown by a non-significant main effect of encoding type, *F*(1, 56) = 0.584, *p* = 0.448, 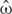
^2^ < 0.001. More importantly, there was no significant practice type × encoding type interaction, *F*(2, 112) = 1.529, *p* = 0.221, 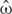
^2^ = 0.006, indicating that the direct testing effect did not reliably differ in size as a function of encoding type.

#### Recall Speed

Consistent with Experiments 1 and 2, there was no main effect of encoding type, *F*(1, 56) = 0.027, *p* = 0.869, 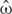
^2^ < 0.001, but a main effect of practice type, *F*(2, 112) = 27.431, *p* < 0.001, 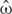
^2^ = 0.139. The latter finding indicates that verb targets were faster retrieved for retrieval-practiced action phrases (*M* = 1.339, *SD* = 0.544) than for action phrases that were restudied (*M* = 1.654, *SD* = 0.828), *W* = 1133.000, *p* < 0.001, *r*_rb_ = 0.558, 95% CI [0.322, 0.729] (one-tailed), or only studied (*M* = 2.010, *SD* = 0.618), *t*(57) = 8.260, *p* < 0.001, *d* = 1.085, 95% CI [0.757, 1.407] (one-tailed). This test-related advantage in recall speed did not differ as a function of encoding type, indicated by a non-significant practice type × encoding type interaction, *F*(2, 112) = 0.904, *p* = 0.408, 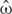
^2^ < 0.001.

To conclude, Experiment 3 provided evidence for the direct testing effect in terms of enhanced recall accuracy, reduced delay-contingent decrements, and accelerated recall speed. Most importantly, we demonstrated a classical direct testing effect via a cross-over interaction, although subjects re-experienced more phrases during restudy practice (100%) than during retrieval practice at the second intermediate test (76.509% [17.059%]), that is, 80.625% (*SD =* 10.807%) for the enacted phrases, and 72.098% (*SD =* 21.211%) for the read-aloud phrases. Notably, the intermediate levels of successful recall significantly varied between experiments, *F*(2, 138) = 16.346, *p* < 0.001, 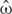
^2^ < 0.179. Tukey’s *post hoc* tests revealed that intermediate recall performance in Experiment 3 was significantly higher than in Experiment 2 (*M* = 52.714; *SD* = 19.378), *p* < 0.001, *d* = 1.325, and marginally higher than in Experiment 1 (*M* = 68.229; *SD* = 22.061), *p* = 0.078, *d* = 0.425, reducing the immediate restudy advantage in Experiment 3. In addition, providing different sets of phrases for the immediate and delayed final memory tests kept the relative difference in memory strength between retrieval and restudy practice, and prolonging the delay from 1 to 2 weeks helped to reverse the immediate restudy advantage into a testing advantage in long-term retention.

## General Discussion

The present study provides evidence that the direct testing effect occurs in memory for actions. Across three experiments, we demonstrated a direct testing effect via a reduced recall decrement over the long delay, while a testing advantage in long-term retention emerged only in Experiment 3. Importantly, a testing advantage in recall speed emerged already after 2 min. This retrieval benefit emerged for verbal as well as for enactive encoding across experiments. In contrast to the direct testing effect, the benefit of enactment, relative to reading aloud, only materialized in increased recall accuracy but not in recall speed or recall decrement over longer delays. These findings were largely independent of recall direction (i.e., noun-cued recall of verbs vs. verb-cued recall of nouns). Taken together, this set of experiments provides evidence that repeated retrieval leads to generalizable benefits in both recall accuracy and speed, while enactment enhanced mainly recall accuracy, suggesting that both techniques may engender in parts different learning benefits.

### Direct Testing Effect

Across all experiments, we obtained evidence in support of a direct testing effect. That is, repeated retrieval mitigated the recall decrement over the long delay more than restudy practice. While repeated restudy produced higher immediate recall accuracy than repeated retrieval, the restudy advantage was reliably reduced after 1 week (Exp. 1 and 2) or even reverted into a test advantage after 2 weeks (Exp. 3). However, as we did not manipulate different practice schedules (SSRR, SRSR, and SSSS) within one experiment, and varied in Experiment 3 more than one aspect (e.g., prolonging the delay, adding an extra study phase) relative to Experiments 1 and 2, we cannot single out one specific factor that accounts for the emergence of the cross-over interaction effect in Experiment 3. Nonetheless, these findings provide new evidence that this direct testing effect confers to fairly novel materials depicting action events under enhanced encoding conditions via verbal production (i.e., saying aloud phrases) and enactment (performing the action events) during study and restudy.

However, these results appear to be inconsistent with previous studies in action memory that did not reveal any testing effect for enacted phrases in terms of recall decrement (Kubik et al., 2014b, 2016). As discussed, methodological factors may in parts moderate these inconsistencies. Importantly, previous studies used an interleaved-testing paradigm that comprised repeated test–restudy cycles and thereby provided additional restudy opportunities following retrieval practice. Although this paradigm may reflect more realistically the affordances in everyday life, it additionally permitted test-potentiated learning of subsequent restudy. That is, testing phrases (specifically when the retrieval attempt was unsuccessful), compared to restudy, enhanced encoding during the subsequent restudy phase. With regards to the recall decrement over the long-term, this influence of (test-)enhanced encoding via (mainly unsuccessful) prior retrieval attempts is still unknown, while the effect of successful retrieval is well replicated (cf. Rowland, 2014). As the present study isolated this direct (from the indirect) test effect (i.e., no test-ensuing restudy opportunities were provided), it provides more conclusive evidence regarding the direct testing effect. However, this methodological factor does not entirely explain why the testing effect did not emerge following enactive encoding, as it appeared under the same conditions following verbal encoding. An additional factor may be that enactment during restudy enhances the encoding processing during restudy, and is therefore a more active control condition for retrieval practice than the typical ineffective restudy conditions by passive rereading (Kornell et al., 2012). As we additionally extended the delay to 2 weeks and enhanced the intermediate recall levels in Experiment 3, we observed a testing effect for enacted phrases in terms of recall decrement over the long delay.

The majority of research examined testing benefits in terms of the *amount* of recalled information (cf. Rowland, 2014); however, only very few studies did so in terms of the *speed* of recalled information—a complimentary measure of memory accessibility (Keresztes et al., 2013; van den Broek et al., 2013; Racsmány et al., 2018). The present results revealed that retrieval-practiced, relative to restudied, phrases led to reduced recall latencies after the short delay, and demonstrated this direct testing effect for the first time following conditions of both verbal encoding (i.e., saying aloud) and enactive encoding (i.e., motoric performance). The present study confirms and extends the few prior study findings showing immediate and delayed testing benefits in recall latencies when subjects silently studied Swahili–Dutch or Swahili–German word pairs (Keresztes et al., 2013; van den Broek et al., 2013). Note that this testing effect on memory accessibility was quite robust, as it was demonstrated by various measures of recall speed that are more or less accurate (last-key submission times, van den Broek et al., 2013; first-key submission times: Keresztes et al., 2013; Racsmány et al., 2018; pressing the space button, the present study).

The results of the present study are largely consistent with the distribution-based bifurcation model. First, the delay-contingent testing effect on recall accuracy resonates with the idea that retrieval-practiced as compared to restudied phrases have on average a higher memory strength. That is, retrieval-practiced phrases stay longer above the recall threshold than restudied phrases with proceeding time even when forgetting rates are similar for both conditions. This explains the finding that the restudy advantage decreased, or even reverted into a testing advantage after the long delay, despite the fact that subjects re-experienced restudied phrases two times more than retrieval-practiced phrases during the learning phase. Notably, only in Experiment 3, we observed a cross-over interaction effect and a testing advantage in long-term retention. From the view of the distribution-based bifurcation model, we can speculate that at least the combination of two factors may have contributed to the potential underestimation of the direct testing effect in Experiments 1 and 2: (i) the relative difference in memory strength was decreased between retrieval-practiced and restudied phrases, as all phrases were tested after the immediate test, enhancing also the memory strength of restudied phrases; (ii) retrieval-practiced items benefited less from the 1-week (compared to the 2 weeks) delay as the less strengthened restudy items would fall more likely below the recall threshold the more memory strength decreases with proceeding time. Second, the immediate (and delayed) testing effect on recall speed in Experiments 1–3 are also consistent with the distribution-based bifurcation model. Given that retrieval-practiced phrases have increased average memory strength for both enacted and read-aloud phrases and recall latencies reflect memory strength, the finding is in line with the distribution-based bifurcation model that retrieval-practiced phrases were faster recalled than restudied phrases independent of delay and encoding type. In sum, the results on the direct testing effect of the present study are largely in line with the distribution-based bifurcation model.

### Enactment Effect

Although not being the main aim of this study, we also reported the results regarding the effect of encoding type on recall accuracy and for the first time on recall speed. Comparing verbally and enactively encoded action phrases reminded us of the enactment benefit on recall accuracy (see Nilsson, 2000; Roediger and Zaromb, 2010). This enactment effect was significantly demonstrated and similarly sized in Experiments 2 (verb-cued recall of nouns; 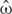
^2^ = 0.107) and Experiment 3 (noun-cued recall of verbs; 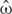
^2^ = 0.110) at the final tests, while also marginally existent and smaller in size in Experiment 1 (noun-cued recall of verbs; *p* = 0.054; 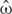
^2^ = 0.057). These results are consonant with previous research findings (Earles and Kersten, 2000; Steffens et al., 2009) and support the idea that enactment may strengthen the verb–noun association in both directions (Kormi-Nouri and Nilsson, 1998; Steffens et al., 2009; Kubik et al., 2016). Based on this notion of enhanced verb–noun relational processing, it may be predicted that enactment also enhances memory accessibility in terms of reducing recall latencies. However, no enactment effect for recall latencies has been shown for both noun- and verb-cued recall. As previous research has not yet reported such enactment-related enhancement of memory accessibility, further research is needed. For example, to our knowledge, no systematic body of research investigated the enactment benefit on memory accessibility across different delays and test formats (but for single findings of shortened recognition latencies after enactment, see Freeman and Ellis, 2003).

Notably, the size of the enactment effect did not vary as a function of delay and practice type. That is, enactive relative to verbal encoding did not reduce the recall decrement over the long delay, and its memorial effect emerged for both restudied and retrieval-practiced action phrases. The parallel recall decrement following both encoding conditions is in line with findings of Nilsson et al. (1989) but is inconsistent with previous findings of our research group, showing a reduced recall decrement following enactment (Kubik et al., 2014b, 2016). The difference in initial recall levels between studies may be one important factor that moderates this results pattern. The relatively high initial recall levels in our previous studies (≥0.85; Kubik et al., 2014b, 2016) may have reduced the enactment effect after the short delay, but not the long delay, thereby leaving the impression that the recall decrement following enactment was reduced. This potential concern was less given in the present study and Nilsson et al. (1989) with initial recall levels for enacted phrases of ≤0.85. We encourage future research to investigate the recall decrement following enactive vs. verbal encoding for more and longer delays that are ideally evaluated at various and matched levels of recall accuracy (cf. Nilsson et al., 1989; Kubik, 2014).

## Conclusion

Our theoretical starting point was the distribution-based bifurcation model, as the latter was most consistent with the majority of prior research findings on the direct testing effect (for a meta-analytic review and evaluation, see Rowland, 2014). In addition, this theoretical framework provides the possibility to explicitly predict the testing effect in both measures of recall accuracy and speed, and to acknowledge the fact that not all retrieval-practiced phrases are reprocessed during retrieval practice, except those that are successfully retrieved. Considering phrases’ distribution of memory strength comes as a theoretical advantage, when studying the retrieval benefits under different encoding activities and recall levels. We note, however, that the effects of practice type and encoding type emerged largely independent from each other, and retrieval (compared to restudy) practice had positive mnemonic effects on both recall accuracy and recall speed, while enactment did not. These rather independent memory benefits conveyed by retrieval and enactment may be accounted for by the distribution-based bifurcation model when presuming that memory strength can increase in a linear fashion and to some extent beyond the measurable levels of memory tests.

Alternatively, if one presumes that memory strength or any other theoretical notion (e.g., cue–target relational processing, cf. Kubik et al., 2014b, 2016) to be a limited resource and the latter is directly measurable in recall accuracy, the present results would not support that both study techniques rely on the same mechanism. Instead, this results pattern would suggest that retrieval practice and enactment largely rely on different mechanisms. Given that enactment elaborates the verb–target relation within action phrases via semantic or motoric mechanisms (Kormi-Nouri and Nilsson, 1998; cf. Zimmer et al., 2001; Steffens et al., 2009), retrieval practice may specifically enhance the encoding of contextual-episodic traces compared to restudy practice that helps learners to discriminate target items against alternative retrieval candidates during memory search (cf. Karpicke et al., 2014; Rowland, 2014). Thus, the present results may be in large also consistent with other theoretical accounts, however, it was not the primary aim to test them against each other, and we remain speculative here. We agree with recent reviews (cf. Rowland, 2014; Kornell and Vaughn, 2016) that the direct testing effect may be determined by multiple memory mechanisms dependent on the experimental conditions.

Given the fact that we often need to remember actions in everyday life, future research should investigate more directly the additional benefits of an enactive test format for retrieval-practice effects. A recent study provided first evidence by manipulating both study techniques commonly during the intermediate tests (Kubik et al., unpublished). In support of the current results, we observed that overtly producing the retrieved responses (e.g., verb targets) by enacting them, compared to covertly (silently) retrieving them during intermediate tests, had a beneficial effect on long-term retention, above and beyond the direct testing benefit. Consistent with the current results, these additive effects provide evidence that enactment and retrieval practice produce rather independent learning benefits. Further research is needed to examine the relative benefits of enactive vs. covert retrieval for the direct testing effect, when manipulating the congruency of these retrieval formats at both intermediate and finals tests. To conclude, the direct testing effect in terms of recall accuracy and recall speed appears to be highly generalizable even with more complex, action-oriented stimulus materials and in the context of effective encoding strategies, such as verbal production and enactment. In comparison, the enactment effect was reliable in recall accuracy, however, did not enhance recall speed nor reduced the recall decrement over the long delay. Thus, retrieval practice and enactment may confer in parts different learning benefits. Future research may determine common and distinctive underpinnings of these learning techniques.

## Ethics Statement

This study was carried out in accordance with the recommendations of the American Psychological Association’s Ethical Principles of Psychologists and Code of Conduct. All subjects gave written informed consent in accordance with the Declaration of Helsinki (2013) before participating in the study, with the understanding that they could quit at any time. The Regional Ethic Review Board, Stockholm (www.epn.se) concluded that there are no ethical concerns regarding the proposed experiments on the testing effect, including the current study, in order to be further reviewed.

## Author Contributions

VK developed the experimental design, carried out data collection and analysis of the experiments of this paper, and drafted and revised the paper. FJ, MK, and WM provided critical comments. All authors unanimously made final approval of the version to be submitted for publication.

## Conflict of Interest Statement

The authors declare that the research was conducted in the absence of any commercial or financial relationships that could be construed as a potential conflict of interest.

## Footnotes

1 The proportional recall decrement was calculated as follows:  (cf. Loftus, 1985; Roediger and Karpicke, 2006a). We also calculated the recall decrement, which led to highly similar results and were implicated in the results on recall accuracy. Henceforth, we used the term *recall decrement* to distinguish it from *forgetting*. Based on the distribution-based bifurcation model, the latter is assumed to be similar for both practice types, while retrieval-practiced phrases should decrease less in recall accuracy (= recall decrement) over the retention interval due to their increased memory strength.

2 The results and conclusions largely remain when calculating mean values of individual median response latencies for Experiment 1. This was also true for Experiments 2 and 3.

3 We restricted the final analyses of Experiments 1–3 to the recall-speed data after 2 min for both theoretical and methodological reasons. Theoretically, the distribution-based bifurcation model predicts an immediate test effect after the short delay. Methodologically, the recall data after 1 week may be (i) influenced by the immediate final test, in which all restudied and retrieval-practiced phrases were tested and (ii) may be prone to stronger item-selection effects due to the lower accuracy levels in long-term retention.

4 https://osf.io/vg4qd/
